# Notes and News

**Published:** 1899-02-18

**Authors:** 


### NOTES AND NEWS.

(Collected and Communicated.)

At the meeting of the Metropolitan Asylums Board last Saturday it was resolved that every architect employed by the Managers should be, as a general rule, engaged under an agreement sealed by the Board, and a draft agreement with an architect submitted by the Works Committee was approved and adopted as the basis of future agreements with architects upon the understanding that the actual agreement should be settled in each case by the Board's solicitors.

The new cottage hospital at Margate, which serves as a Jubilee memorial, has just been opened by the Right Hon. James Lowther, M.P. The idea was initiated by Mr. Bertram Thornton, who, having rendered many years' valuable service on the medical staff of the old hospital, was well aware of the necessity for a more convenient building. Of the old building, the Wilcox and Lucas wards and the operating-room alone remain, and the rest of the hospital has given place to a large and complete modern building. The accommodation for the staff is now placed on a proper basis, and six more beds having been added, 20 patients can now be accommodated. The cost of the reconstruction has been £1,600.

Before the agitation has ceased amongst anti-vaccinationists in regard to the action of the Peabody Trustees in demanding that all dwellers in their buildings shall be vaccinated, another serious grievance has arisen. This time the Admiralty are the offenders. They have decreed that unvaccinated families of marines are not to be allowed to live in barracks, nor are they to be conveyed to any foreign station at public expense, nor may the children attend the divisional schools. Whilst the "unconscientious" will have cause to rejoice at the manner in which the Admiralty is protecting its employés from a serious danger, the anti-vaccination agitator will have a fine example of "coercion" to quote. The Admiralty are, however, strong enough to set an example which we hope will be followed in many directions.

A new workhouse infirmary for 164 patients has been erected at Wakefield. The estimated cost is about £37,000.

At the recent annual meeting of the Liverpool Hospital for Women, the proposal to extend the accommodation of the institution was warmly supported.

An interesting account of the visit of the Emperor and Empress to Palestine appears in the new edition of "Cook's Excursionist" for February.

A fire broke out last week in the small-pox ward of the Islington Infirmary. Much alarm was caused, but the patients were safely removed. Considerable damage was done to the roof of the building.

A scheme for the adoption of the open-air treatment for consumption is being considered at the Brompton Hospital for Consumption. A proposal to establish a sanitorium in the country in connection with the hospital is also under consideration.

Mrs. Spender is once more actively interested in the cause of the London Hospital. The object she now has in view is the establishment of a convalescent home for the use of patients from the hospital. It is desired to raise a fund for the furnishing and maintenance of the proposed institution; £500 is asked for preliminary expenses, and £800 per annum for maintenance. An anonymous friend has promised £250 for five years, and a suitable house on the Kentish coast has been found. A ball in aid of the project will be held on April 20th at the Hotel Cecil. The patients of the London Hospital come from such poor homes, and the scheme is so obviously a necessary one, that we feel sure it will meet the support it merits.

The day is rapidly passing when officials and others responsible for the management of institutions are content to proceed without periodical inquiry as to the methods pursued in other establishments. Much may be learnt by comparing methods and results, and, realising this, the officials who are genuinely interested and desire efficiency in their work gladly welcome scrutiny and comparison as to the manner in which their institutions are conducted. The proceedings at the last annual meeting of the East Suffolk and Ipswich Hospital, published in the *Ipswich and East Anglian Times*, were most instructive, and strikingly illustrate the intelligent spirit of interest in the conduct of hospitals which is now showing itself. At this meeting Mr. Herbert Mason, taking advantage of the opportunity afforded by the comparative tables published in Burdett's "Hospitals and Charities," was able to show in what way the Ipswich Hospital compared with other institutions of the same description. The comparison was a happy one for Ipswich, of course, and although we cannot expect hospital managers to make these useful statistics public when they tell against their own institutions, still a useful spirit of emulation will stir them to remove unpleasant differences which show so relentlessly in tabulated form. The uniform system of accounts is largely to thank for facilitating useful investigations.

